# Experimental Identification of Statistical Correlation between Mechanical Properties of FRP Composite

**DOI:** 10.3390/ma13030674

**Published:** 2020-02-03

**Authors:** Shufeng Zhang, Tongzhen Xing, Haibin Zhu, Xun Chen

**Affiliations:** 1 Science and Technology on Integrated Logistics Support Laboratory, National University of Defense Technology, Changsha 410073, China; sfzhang@nudt.edu.cn (S.Z.); chenxun@nudt.edu.cn (X.C.); 2 School of Aerospace Engineering, Beijing Institute of Technology, Beijing 100811, China; 3120170014@bit.edu.cn; 3 Flexible Optical Measurement and Technology Centre, Institute of Flexible Electronics Technology of Tsinghua University, Jiaxing 314000, China

**Keywords:** composite, statistical correlation, mechanical property, uncertainty

## Abstract

Recent prediction on the heavy statistical correlation between the mechanical properties of fiber reinforced composite (FRP) raises new concerns on the accurate reliability evaluation of composite structures, but such statistical correlation still lacks experimental verification. In this work, an experimental methodology is proposed to determine the statistical correlation between mechanical properties of unidirectional FRP composite. A rectangular shaped carbon fiber reinforced plastic (CFRP) specimen with a circular hole is loaded by tension, and 3D digital image correlation (DIC) is employed to characterize the heterogeneous strain field around the hole. Virtual field method (VFM) is used to derive *E*_11_, *E*_22_, *ν*_12_, and *G*_12_ of specimens. Specimen configuration considering fiber angle and hole diameter is optimized to achieve accurate determination of correlation coefficients. Experimental results on the linear correlation coefficients between *E*_11_, *E*_22_, *ν*_12_, and *G*_12_ agree well with previous theoretical predictions.

## 1. Introduction

Fiber reinforced plastic (FRP) composite is intrinsically a multiphase and heterogeneous material, and significant uncertainty has been observed on its mechanical properties [1,2,3]. Therefore, it is essential to investigate the behavior of FRP structures in probabilistic manners [4,5,6,7], which allows evaluation of structure reliability or failure probability by incorporating uncertainties. Most of the relevant studies have calculated FRP structure reliability by considering ply-scale (or lamina-scale) uncertainty such as ply mechanical properties, ply thickness, and ply angle [8,9,10,11] (to mention just a few), where ply mechanical properties are often considered to be random variables independent from one another. Recent theoretical studies by Shaw et al. [12] suggest that ply mechanical properties would be statistically correlated, and Zhang et al. [13,14,15] further demonstrate that the statistical correlation between ply in-plane elastic properties could heavily affect structure reliability. However, to the authors’ knowledge, the theoretically predicted statistical correlation between ply mechanical properties has not been experimentally verified, and the statistics on the mechanical properties of fiber and matrix employed for the prediction of ply-scale statistical correlation include many assumptions. This raises a problem surrounding how much we can trust the statistical correlation predicted from micro-scale constituent properties. Conventional macro-scale test methods on measuring FRP mechanical properties suggested by (American Society for Testing and Materials) ASTM or (International Organization for Standardization) ISO [16,17,18,19] only provide a certain modulus for a test configuration or a specimen, which provides no information on the statistical correlation between different moduli. This may be a major reason why ply elastic properties are often considered to be statistically independent of one another in most current studies on reliability evaluation of composite structures.

Several inverse methods, such as finite element model updating method [20], constitutive equation gap method [21], and virtual fields method (VFM) [22], have been recently developed to derive all constitutive parameters of engineering materials from one test configuration and one specimen. The VFM is an efficient technique to characterize material properties, which avoids iterative problem solutions and requires little computation effort. Pierron et al. [23] employ a combination of VFM and speckled interferometry to achieve elastic properties of unidirectional glass–epoxy composite, using the Iosipescu test configuration. Wang et al. [24] employ a combination of VFM and DIC (digital image correlation [25,26]) to characterize elastic properties of orthotropic foam material. The VFM is also used to identify dynamic orthotropic parameters of composites through ultra-high-speed imaging and grid method [27,28,29]. 

The objective of this work is to experimentally identify the statistical correlation between in-plane elastic properties of unidirectional FRP. Carbon fiber reinforced plastic (CFRP) specimens are fabricated and a circular hole is cut out in the specimen central region. Hole size and fiber angle of the specimen are optimized to achieve accurate determination of specimen elastic properties. DIC is adopted to characterize nonuniform strain field around the hole, and the VFM is used to derive *E*_11_, *E*_22_, *ν*_12_, and *G*_12_ of specimens. A total number of 15 specimens are tested, and linear correlation coefficients between *E*_11_, *E*_22_, *ν*_12_, and *G*_12_ are determined. Experimental results show good agreement with previous theoretical prediction on the statistical correlation between CFRP elastic properties.

## 2. Experimental Arrangement

**Figure 1:**
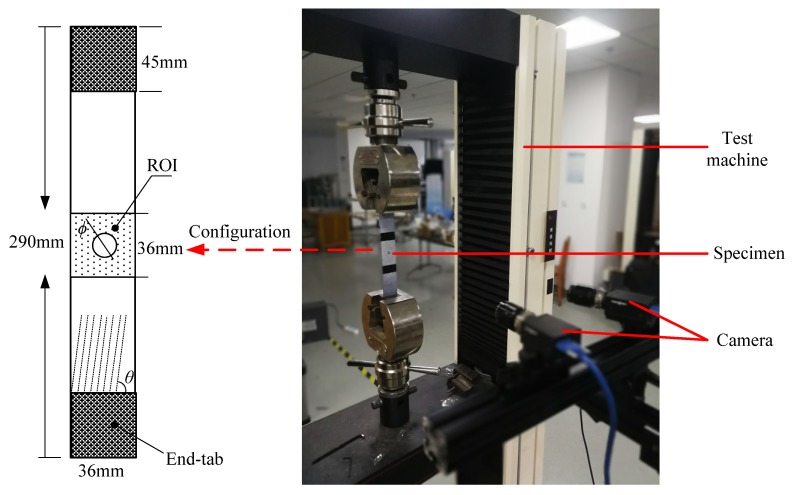
Specimen configuration and experimental set-up.

The experimental apparatus and specimen configuration used in this work are shown in Figure 1. The material studied in this work is CFRP with a configuration of T300/Epoxy. Unidirectional CFRP specimens with dimension of 290 mm × 36 mm (length × width) were cut from a large composite panel made by prepreg molding. The composite panel is made of 12 layers of prepreg, and its nominal thickness is 1.5 mm. A circular hole was cut at the center of the specimen. In practice, to ensure high enough measurement accuracy, a region of interest (ROI) is defined as a square area with a size of 36 mm × 36 mm around the hole, as shown in the dashed square marked in Figure 1. A specimen with such a shape configuration would introduce heterogeneous stress state around the hole, which is required to determine *E*_11_, *E*_22_, *ν*_12_, and *G*_12_ by the VFM. Fiber angle (*θ*) and hole diameter (*ϕ*) are to be optimized so that the elastic properties derived by the VFM are least sensitive to strain measurement error. The specimen was fixed between two grips mounted on an electromechanical test machine with a load capacity of 50 kN. Tensile load was applied by moving the actuator of the test machine at a speed of 1 mm/min until an initial crack occurs around the hole, which generated a clear burst noise. The very low speed of load introduction enables the specimens to be subjected to quasi-static loading condition. 

Three dimensional DIC was employed to characterize nonuniform deformation of specimens. The fundamental principle of DIC is to calculate full-field displacement and strain by tracking specific speckle patterns in digital images obtained from an objective. To form speckle patterns, all of the specimens in this study were first painted by a layer of white coating and then sprinkled with black particles. Images of specimen surface were recorded synchronously with the tensile load data read from a load cell mounted on the test machine. Commercially available software Vic-3D (provided by Correlated Solutions Inc., Irmo, SC, USA) was used to calculate full-field strain of specimens, and details on the DIC set-up are listed in Table 1. The deformation field was calculated by employing first-order shape function and criteria of sum of squared difference (SSD). Cubic b-spline interpolation and binomial low-pass filter were selected to reduce noise on displacement measurement, and strain was calculated from the displacement field after being spatially smoothed with a window size of 15 pixels. Such a configuration is considered to be capable of providing a strain field with good accuracy [30]. As possible misalignment between grips would introduce out-of-plane motion, 3D DIC was employed so that possible out-of-plane motion could be compensated in strain calculation. 

## 3. Virtual Fields Method (VFM)

The VFM is essentially based on the principle of virtual work [22,31]. The general equation of the principle of virtual work can be expressed as:

(1) $$-\int_{V_{m}}\underline{\underline{\sigma }}:\underline{\underline{\varepsilon^{*}}}dV+\int_{\partial Vm}\vec{T}.\;{\vec{u}}^{*}dS=\int_{Vm}\rho \vec{a}.\;{\vec{u}}^{*}dV$$

where $\underline{\underline{\sigma }}$.is the Cauchy stress tensor, $\vec{T}$ the Cauchy stress vector acting at the boundary surface $\partial V_{m}$, $\vec{a}$ is the acceleration vector over the volume $V_{m}$, ${\vec{u}}^{*}$ is a zero-order vectorial function referred to as “ virtual displacement field”, $\varepsilon^{*}$ is the virtual strain tensor derived from ${\vec{u}}^{*}$ and ρ is the materials density. In Equation (1), the first item at the left hand side of the equal sign is ‘internal virtual work’, the second item at the left hand side of the equal sign is ‘external virtual work’, and the item at the right hand side of the equal sign is ‘virtual work’ done by acceleration fields. Under quasi-static conditions, the item at the right-hand side of the equal sign is null.

The basis of VFM is to exploit Equation (1) with particular virtual fields. In the case of linear elasticity, elastic parameters can be identified directly from a linear system which is built up through rewriting Equation (1) with as many independent virtual fields as unknowns, provided that the measured kinematic fields are heterogeneous. It is worth emphasizing that most full-field measurement techniques only provide deformation over the external surface of the solid. Therefore, specimens need to be well designed so that the surface response is respective of the volume response. Typically, a thin plate under plane stress assumption is usually employed. In the case of an in-plane test, if *h* is the thickness of the volume *V_m_* and *S* is the associated planar surface, for the quasi-static condition investigated in this work, Equation (1) reduces to a 2D situation as:

(2) $$h\int_{S}\underline{\underline{\sigma }}:\underline{\underline{\varepsilon^{*}}}dS=\int_{S}\vec{T}.\;{\vec{u}}^{*}dL$$

If a plane stress is applied on an orthotropic material, according to Hooke’s Law, the relationship between Cauchy stress and linear strain in the material coordinate system can be expressed by:

(3) $$\left\{\begin{array}{l} \sigma_{11}\\ \sigma_{22}\\ \sigma_{12} \end{array}\right\}=\left[\begin{array}{lll} Q_{11} & Q_{12} & 0\\ Q_{21} & Q_{22} & 0\\ 0 & 0 & Q_{66} \end{array}\right]\left\{\begin{array}{l} \varepsilon_{11}\\ \varepsilon_{22}\\ 2\varepsilon_{12} \end{array}\right\}$$

where *Q_ij_* are the stiffness matrix components in the material coordinate system, $\sigma_{i}$ and $\varepsilon_{i}$ are the stress and strain components, and subscripts 1, 2, and 6 represent the longitudinal, transverse, and in-plane shear components, respectively. 2*ε*_12_ is equivalent to *γ*_12_, which is the shear strain in 1-2 plane. For unidirectional FRP, *Q_ij_* is expressed by:

(4) $$\begin{array}{l} Q_{11}=\frac{E_{11}}{1-\nu_{12}^{2}(E_{22}/E_{11})}\\ Q_{22}=\frac{E_{22}}{1-\nu_{12}^{2}(E_{22}/E_{11})}\\ Q_{12}=Q_{21}=\frac{\nu_{12}E_{11}}{1-\nu_{12}^{2}(E_{22}/E_{11})}\\ Q_{66}=G_{12} \end{array}$$

Since the CFRP specimen might be prepared with an off-axis angle *θ*, the strain characterized in a reference system (denoted by *x*-*y*) needs to be firstly transformed to material coordinate system (denoted by 1-2) by Equation (5) and is then substituted in Equation (2) to derive elastic properties of specimens.

(5) $$\left\{\begin{array}{l} \varepsilon_{11}\\ \varepsilon_{22}\\ \varepsilon_{12} \end{array}\right\}=\left[\begin{array}{lll} \cos^{2}\theta & \sin^{2}\theta & 2\cos\theta \sin\theta \\ \sin^{2}\theta & \cos^{2}\theta & -2\cos\theta \sin\theta \\ -2\cos\theta \sin\theta & 2\cos\theta \sin\theta & 2(\cos^{2}\theta -\sin^{2}\theta )\; \end{array}\right]\left\{\begin{array}{l} \varepsilon_{x}\\ \varepsilon_{y}\\ \varepsilon_{xy} \end{array}\right\}$$

To identify 4 independent stiffness components in Equation (4) using the virtual fields method, it is necessary to define four independent virtual fields (u*) with respected to the required boundary conditions. In the present case, since only the longitudinal resultant force is introduced in the present work, the virtual longitudinal and transverse displacement along the bottom edge of ROI and the virtual transverse displacement along the top edge of ROI are zero, and the virtual longitudinal displacement along the top edge of ROI is a certain constant.

## 4. Optimization of Specimen Configuration

To achieve accurate determination of *E*_11_, *E*_22_, *ν*_12_, and *G*_12_ of unidirectional CFRP by the VFM, it generally needs to generate a heterogeneous strain/stress field which contains recognizable deformation components corresponding to all the four elastic properties. For the specimen used in this work, the fiber angle *θ* and hole diameter *ϕ* would affect the heterogeneous deformation status around the hole, and they consequently affect the accuracy of measurement of *E*_11_, *E*_22_, *ν*_12_, and *G*_12_. Considering the main error source for the determination of *E*_11_, *E*_22_, *ν*_12_, and *G*_12_ is random error of strain measurement by DIC, an optimized specimen configuration should enable the determination of specimen elastic properties to be mostly insensitive to the random error of strain measurement. 

To achieve optimized specimen configuration which is mostly insensitive to random error of strain measurement, specimen configurations with the hole diameter (*ϕ*) ranging from 6–8 mm and the fiber angle (*θ*) ranging from 70°–90° were investigated. The selected ranges are appropriate according to the results obtained and discussed later. Linear–elastic 2D finite element (FE) modeling was first conducted to investigate nonuniform strain field around the hole by commercially available software ANSYS Ver14.5 (provided by ANSYS Inc., Canonsburg, PA, USA). As the specimen thickness is much smaller than the width and length, assumption of plane stress state would be reasonable. Four-node orthotropic shell element (shell181) was employed to build the FE model, and elastic properties used for the element are shown in Table 2 [1]. The elastic properties of T300 carbon/epoxy shown in Table 2 are experimentally obtained following methodologies recommended by ASTM standard, and a total number of 70 specimens were employed for the tensile and shear test. As the specimen used in this work is also made of T300 carbon fiber and epoxy, values in Table 2 would be a good reference for constructing FE models aiming for specimen optimization. 

**Figure 2:**
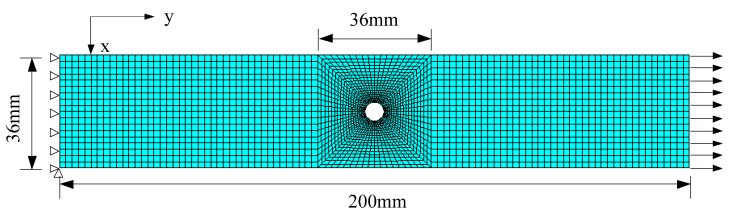
Geometrical size and mesh of finite element (FE) model.

The geometrical size and mesh of the FE model are shown in Figure 2. To get an accurate nonuniform strain field around the hole, fine and mapping mesh is generated in ROI in this work. An elaborate mesh convergence study concerning the average strain over the ROI was obtained. Elements at the edge of the hole were set at a size of around 0.6 mm × 0.8 mm (the edge of the circle is meshed to identical 36 elements), and element size increases as the element moves away from the hole. Elements out of ROI were set at a size of 2 mm × 2 mm. As mapping mesh is achieved, the element size in the ROI changes slightly due to the variation of hole diameters from 6 mm to 8 mm, but the variation has negligible influence on mesh convergence. Such mesh approach is considered to be appropriate to represent nonuniform stress/strain state around the hole [32].

**Figure 3:**
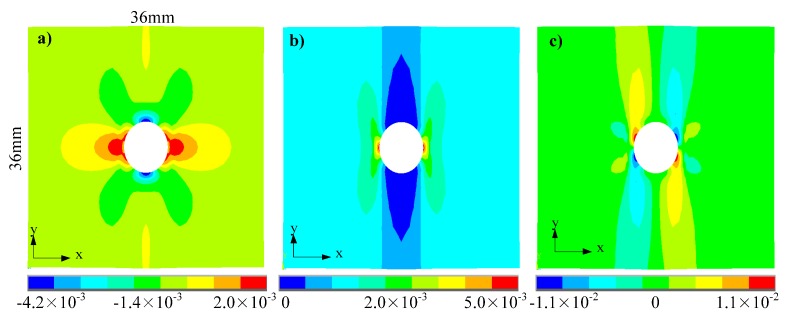
Strain distribution derived from FE model while hole diameter *ϕ* = 6 mm and fiber angle *θ* = 90°: (**a**) *ε_x_*; (**b**) *ε_y_*; (**c**) *γ_xy._*

Nodes on the left end of the geometrical model are constrained in the y-direction, and nodes on the right end bear uniformly distributed tensile load at a total value of 10 kN. From experimental observation, tensile load around 10 kN would trigger initial crack for specimens with considered fiber angles and hole diameters. Figure 3 shows the nonuniform strain field derived from the FE modeling (*θ* = 90° and *ϕ* = 6 mm), where only deformation in the ROI is considered in this work. Figure 3a plots x-direction strain, showing that significant x-direction compressive deformation exists near the top and bottom areas of the hole; Figure 3b plots y-direction strain, showing that significant y-direction tensile deformation exists near the left and right areas of the hole; Figure 3c plots shear strain in the x-y plane, showing that significant in-plane shear deformation exists near the top-right, top-left, bottom-right, and bottom-left areas of the hole. 

**Figure 4:**
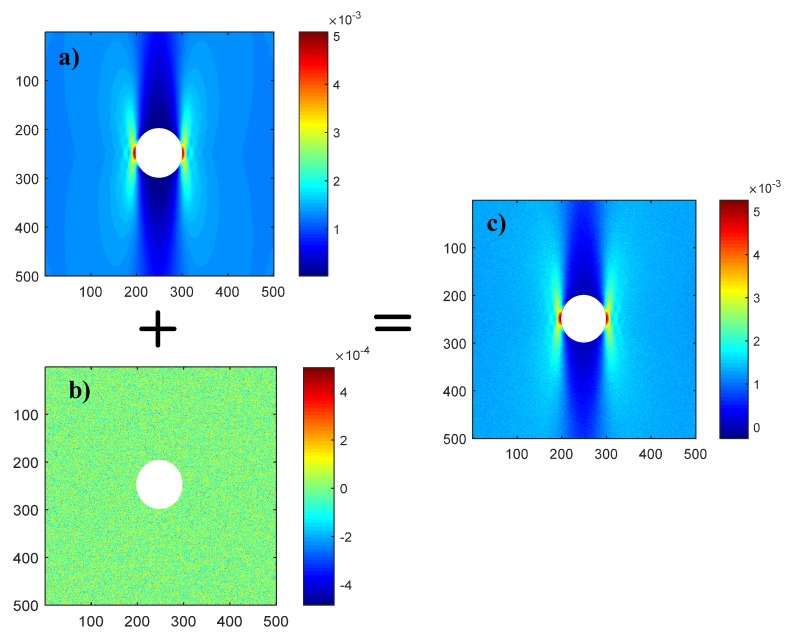
Process of adding random error field on strain field (*ε_y_*) derived by FE: (**a**) strain field derived from FE; (**b**) a typical sample of random strain field sampled from Gaussian distribution N(0, 1 × 10^−4^); (**c**) superposition of Figure (**a**) and Figure (**b**).

**Figure 5:**
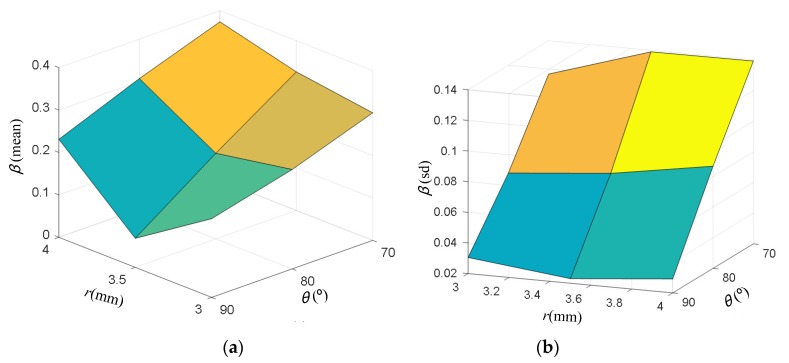
Error function *β*: (**a**) mean value; (**b**) standard deviation.

To simulate the effect of limited strain accuracy after the actual strain measurement by DIC, random strain field sampled from Gaussian distribution N (0.1 × 10^−4^) was added on the strain field (*ε_x_*, *ε_y_*, and *γ_xy_*) derived by FE modeling, as illustrated in Figure 4. The raw strain shown in Figure 4a at element centroid and the related coordinate of these irregular elements in the ROI were first output from ANSYS. A regular strain field consisting of 500 by 500 data points was then produced from the raw strain and coordinate through interpolation (interpolation function named griddata with the type of cubic in MATLAB was employed in the present work). The random strain field shown in Figure 4b mimics strain measurement noise by DIC, and such strain measurement noise would consequently lead to errors on the determination of CFRP elastic properties. Since noise of the strain measurement of DIC also depends on specimen speckle quality, which varies slightly between different specimens, strain noise at slightly larger magnitude than strain resolution shown in Table 1 was employed. To evaluate the effect of hole-diameter and fiber-angle in the elastic property derived by the VFM method, an error function beta is defined as:

(6) $$\beta =\frac{1}{4}\sum_{i=1}^{4}\frac{\left|\Delta_{i}-\Delta_{i-ref}\right|}{\Delta_{i-ref}}$$

where Δ*_i_* denotes elastic property derived by the VFM method, and Δ*_i-ref_* denotes the reference elastic property (elastic property used in the FE modeling). *β* represents a difference between elastic properties obtained using noisy strain contour and the reference values. A smaller *β* indicates better accuracy on the determination of CFRP elastic properties, and vice versa. For each specimen configuration, 1 × 10^4^ samples of random strain field were drawn to add to the strain field derived by FE to calculate *β*. A typical sample of random strain field is shown in Figure 4b. Results on the statistics of *β* for different specimen configurations are shown in Figure 5. Figure 5 clearly shows that specimen configuration with *ϕ* = 7 mm and θ = 90º provides the best prediction on the elastic properties considering both the mean value and standard deviation of *β*, and this geometrical configuration is employed for specimens in the following experimental study. 

## 5. Influence of Strain along Hole Edge on Measurement of Correlation Coefficients

In practical strain measurement using the subset-based DIC, strain in the subsets along the very edge of the hole is missed due to the intrinsic nature of strain calculation algorithm, which might affect the identification of parameter identification [33]. To investigate the effect of the strain missing at the very edge of the hole, a comparison was conducted on deriving the CFRP elastic properties using the strain contour derived by FE modeling in 2 cases, with and without the strain values of the elements along the edge of the hole. 

One thousand groups of the CFRP elastic properties (*E*_11_, *E*_22_, *ν*_12_, and *G*_12_) were firstly sampled according to statistics in our previous theoretical work [13]. To account for their correlation, Gibbs sampling method was employed, with the procedure as follows [34]:

(1) Initiation of random variable *x_i_* (i = 1, 2,…, n);
(2) For t = 0,1,2,…, do the iterative sampling as follows:
  1. $x_{1}^{\left(t+1\right)}∼p(x_{1}{|x}_{2}^{t},x_{3}^{t},\cdots x_{n}^{t})$
  2. $x_{2}^{t+1}∼p(x_{2}{|x}_{1}^{t+1},x_{3}^{t},\cdots x_{n}^{t})$
  3. ⋯⋯⋯
  4. $x_{j}^{t+1}∼p(x_{j}{|x}_{1}^{t+1},\cdots ,x_{j-1}^{t+1},x_{j+1}^{t}\cdots x_{n}^{t})$
  5. ⋯⋯⋯
  6. $x_{n}^{t+1}∼p(x_{n}{|x}_{1}^{t+1},x_{2}^{t+1},\cdots x_{n-1}^{t+1})$

**Figure 6:**
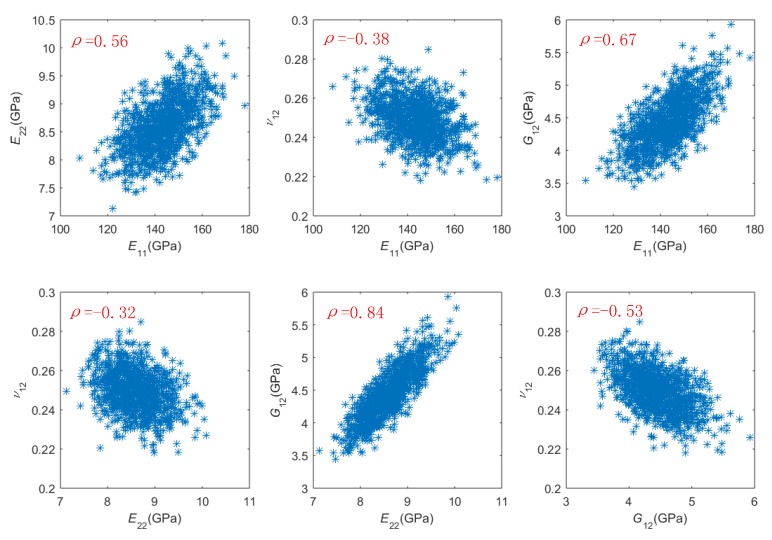
One thousand group of samples on *E*_11_, *E*_22_, *ν*_12_, and *G*_12._

The Gibbs sampling method conducts the sampling process by constructing a Markov chain that has the desired distribution as its equilibrium distribution. Since samples from the beginning of the chain may not accurately represent the desired distribution, samples numbered from 1001 to 2000 were used as the sampling result in this work. The 1000 samples on CFRP elastic properties are shown in Figure 6. Based on the 1000 samples, linear correlation coefficients between CFRP elastic properties were estimated by Equation (7):

(7) $$\rho (x,y)=\frac{Cov(x,y)}{\sqrt{Var(x)Var(y)}}$$

where *ρ*(*x*,*y*) denotes the linear correlation coefficient between random variables *x* and *y*, Cov(*x*,*y*) denotes the covariance of *x* and *y*, and Var(*x*) denotes the variance of *x*. The estimated linear correlation coefficients from the 1000 samples are shown in Figure 6, and they are very close (with a different less than 0.05 on absolute value) to their corresponding reference values given in [13].

**Figure 7:**
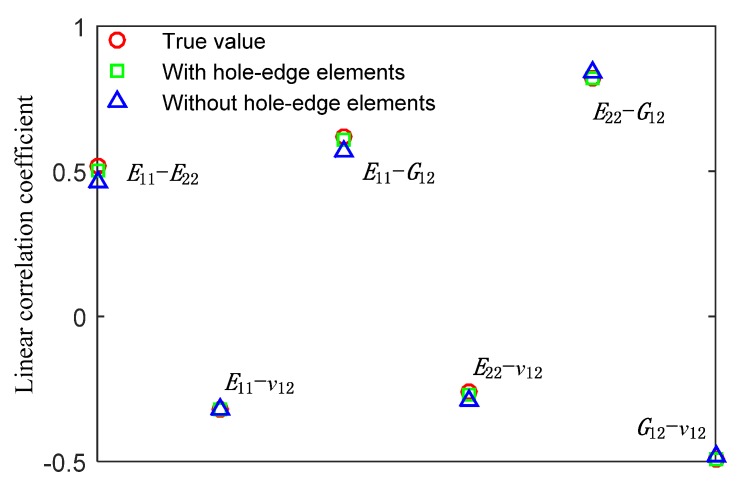
Linear correlation coefficients between carbon fiber reinforced plastic (CFRP) elastic properties.

The 1000 group of sampled elastic properties were then employed to build 1000 FE models similarly as shown in Figure 2 but with specimen configuration with *ϕ* = 7 mm and θ = 90° (configuration for practical specimens), and each FE model provides a strain distribution similar to that shown in Figure 3. The VFM was then employed to derive CFRP elastic properties from strain contour of the 1000 FE models, by considering and not considering strain of the elements at the edge of the hole. Linear correlation coefficients between CFRP elastic properties are then derived using Equation (6). The derived linear correlation coefficients are shown in Figure 7. It is clearly seen that neglecting strain on elements at the very edge of the hole provides very similar linear correlation coefficients to the scenario where strain of all elements is considered, and both scenarios provide linear correlation coefficients very close to the corresponding true values, with a discrepancy smaller than 0.3. Therefore, it is shown that the strain measurement missing at the very edge of hole would have negligible effect on determination of linear correlation coefficients between CFRP elastic properties.

## 6. Experimental Results

**Figure 8:**
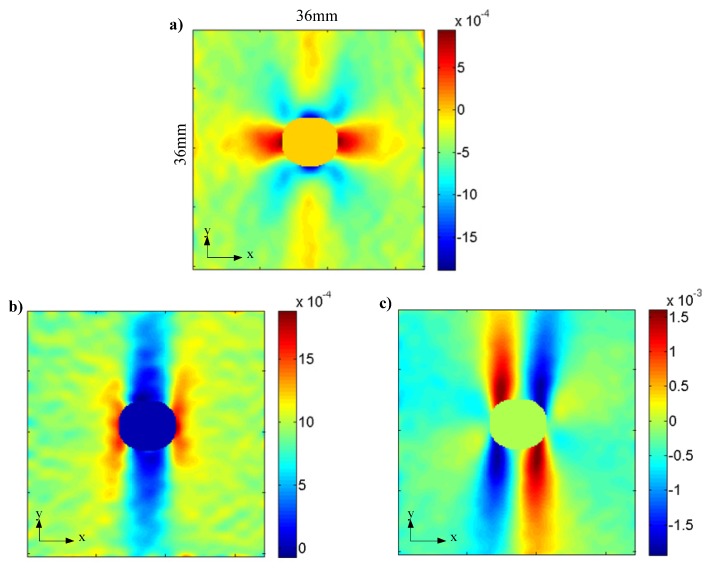
Typical specimen strain distribution measured by DIC: (**a**) *ε_x_*; (**b**) *ε_y_*; (**c**) *γ_xy._*

In this work, a total number of 15 CFRP specimens with the selected dimensions (see Section 4) were tested following the procedure as introduced in Section 2. A typical heterogeneous strain of CFRP specimens measured by DIC is shown Figure 8, where the corresponding load is around 8 kN. It can be seen that the strain patterns of *ε_x_*, *ε_y_*, and *γ_xy_* generally agree well with those derived by FE modeling shown in Figure 3. *E*_11_, *E*_22_, *ν*_12_, and *G*_12_ were then derived for each specimen using the VFM. The statistics including mean value, coefficient of variance (CoV), and linear correlation coefficients are derived from the elastic properties of the 15 specimens, with the results listed in Table 3. It can be seen that the mean values of the elastic properties generally agree well with Jeong and Shenoi [1] where tests are conducted following ASTM standards, though *E*_22_ and *G*_12_ derived by this study are around 20% smaller. The difference between the *E*_22_ and *G*_12_ in this work and Jeong and Shenoi [1] is probably caused by the different types of epoxy used for specimens in this work and Jeong and Shenoi [1], as *E*_22_ and *G*_12_ are mainly dependent on matrix properties.

Very importantly, Table 3 shows that the linear correlation coefficients derived from experiments generally agree well with theoretical prediction in Zhang et al. [13], especially in the following aspects: 

(1) Both the experimental measurement and theoretical prediction show that the linear correlation coefficients between *ν*_12_ and other elastic properties are negative, while the linear correlation coefficients between *E*_11_, *E*_22_, and *G*_12_ are positive;

(2) Both the experimental measurement and theoretical prediction show that significant statistical correlation exists between *E*_22_ and *G*_12_, and this phenomenon would be explained by the fact that they both heavily depend on matrix properties;

(3) Both the experimental measurement and theoretical prediction show that medium correlation exists between *E*_11_ and *E*_22_ (or *G*_12_). 

It is important to notice that the linear correlation coefficients shown in Zhang et al. [13] are derived by assuming a CoV of 5% on fiber volume ratio (FVR). In practice, the variance of FVR depends on different fabrication approaches and even different manufacturers. The difference in the values of the linear correlation coefficients between this experimental work and the theoretical prediction might be attributed to the difference in CoV of FVR between practical specimens used in this work and assumptiona in [13], as it is demonstrated that the CoV of FVR would have a significant influence on the statistical correlation between CFRP elastic properties [13]. Following the methodology shown in [13], if the CoV of FVR lowers to 3%, the predicted linear correlation coefficients between *E*_11_ and *E*_22_, *E*_11_ and *ν*_12_, *E*_11_ and *G*_12_ are 0.31, −0.15, 0.40 respectively, which agree better with the experimental observation.

## 7. Conclusions

An experimental methodology is proposed to determine the statistical correlation between mechanical properties of unidirectional FRP composite, by a combination of DIC and VFM. For the CFRP specimens used in this work, the experimentally measured linear correlation coefficients agree well with theoretical prediction in our previous work [13]. To the best of the authors’ knowledge, this work provides the first experimental verification of statistical correlation between mechanical properties of unidirectional CFRP composite, and the statistical correlation between some elastic properties is indeed significant. Hence, we strongly suggest that designers of composite structures would carefully take into account the statistical correlation between ply elastic properties if structure reliability is required to be accurately evaluated. Our future work will further identify statistical correlation between mechanical properties of FRP made by different constituent materials and different fabrication approaches, and the dependence of the correlation coefficients on constituent-level uncertainty is also to be experimentally studied.

## Figures and Tables

**Table 1:** Details on digital image correlation (DIC) set-up.

Technique Used	Image Correlation
Subset size	31 × 31 pixels^2^
Shift	5 pixels
Camera	8 bit, Vic 5M
Field of view	35 mm (L) × 40 mm (W)
Image recording rate	2 Hz
Spatial resolution	0.0285 mm/pixel
Displacement resolution	around 0.025 pixel
Strain resolution	around 0.8 × 10^−4^

**Table 2:** Elastic properties of shell element in FE model [1].

Properties	*E*_11_	*E*_22_	*ν*_12_	*G*_12_
Values	133.9 GPa	8.84 GPa	0.33	4.45 GPa

**Table 3:** Statistics of unidirectional CFRP elastic properties derived from experiments.

Random Variables	Linear Correlation Coefficient				Mean	CoV	Ref. [1]
	*E*_11_	*E*_22_	*ν*_12_	*G*_12_			
*E*_11_(GPa)	1				135.1	0.043	133.9
*E*_22_(GPa)	0.28/0.54 *	1			7.50	0.145	8.84
*ν*_12_	−0.10/−0.35 *	−0.56/−0.30 *	1		0.33	0.049	0.34
*G*_12_(GPa)	0.31/0.63 *	0.87/0.84 *	−0.76/−0.50 *	1	3.65	0.156	4.45

*: Values by theoretical prediction in Zhang et al. [13]
